# Sulphur- and Selenium-for-Oxygen Replacement as a Strategy to Obtain Dual Type I/Type II Photosensitizers for Photodynamic Therapy

**DOI:** 10.3390/molecules28073153

**Published:** 2023-04-01

**Authors:** Mario Prejanò, Marta Erminia Alberto, Bruna Clara De Simone, Tiziana Marino, Marirosa Toscano, Nino Russo

**Affiliations:** Dipartimento di Chimica e Tecnologie Chimiche, Università della Calabria, 87036 Arcavacata di Rende, CS, Italy

**Keywords:** PDT, TDDFT, Type I/Type II

## Abstract

The effect on the photophysical properties of sulfur- and selenium-for-oxygen replacement in the skeleton of the oxo-4-dimethylaminonaphthalimide molecule (DMNP) has been explored at the density functional (DFT) level of theory. Structural parameters, excitation energies, singlet–triplet energy gaps (ΔE_S-T_), and spin–orbit coupling constants (SOC) have been computed. The determined SOCs indicate an enhanced probability of intersystem crossing (ISC) in both the thio- and seleno-derivatives (SDMNP and SeDMNP, respectively) and, consequently, an enhancement of the singlet oxygen quantum yields. Inspection of Type I reactions reveals that the electron transfer mechanisms leading to the generation of superoxide is feasible for all the compounds, suggesting a dual Type I/Type II activity.

## 1. Introduction

The search for new therapeutic strategies with an enhanced spatial and temporal control of drug activation has significantly boosted scientific interest in Photodynamic Therapy (PDT) [1,2,3]. The latter is a minimally invasive protocol in which biological damage is promoted by the production of highly reactive oxygen species (ROS) or by the in situ generation of singlet oxygen (^1^O_2_), through the so-called Type I and II photoreactions, respectively [4]. Although cancer treatment is definitely the most challenging field of the application of PDT [5,6,7], it is successfully used for a number of different diseases, including cardiovascular disorders [8], bacterial, fungal, and viral infections [9,10,11], rheumatoid arthritis [12], cutaneous manifestations [13], and dental caries [14]. Furthermore, photo-induced ^1^O_2_ utilization for environmental applications such as water purification and disinfections is also emerging as a promising strategy for photocatalysis [15,16,17].

The fewer side effects of PDT, and its higher selectivity compared with classical surgery, are further supported by an invoked immune response that causes a mixture of necrotic and apoptotic cell death, also preventing far-off metastases and tumour recurrence [18,19,20].

**Figure 1:**
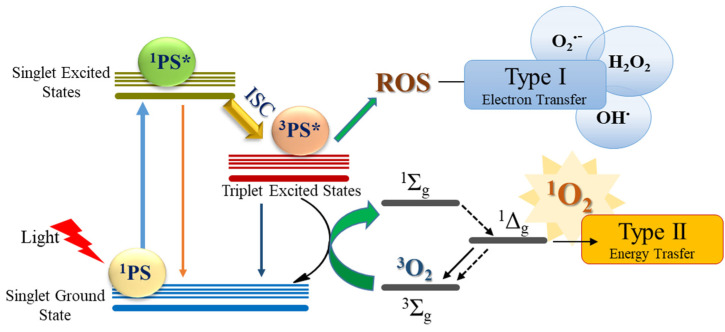
Schematic Jablonski’s diagram showing Type I and Type II photoreactions. In the picture, * stands for excited species.

As a consequence, the design of efficient, light-responsive compounds has significantly increased in the last decades and made it possible to develop new and more effective photosensitizers [21,22,23,24,25], which are also able to overcome some of the most important limitations of current PDT, such as poor light penetration and hypoxia [26,27,28,29,30]. The working mechanism of PDT is sketched in Figure 1.

After administration and localization on a target tissue, the photosensitizer is irradiated by a proper light source whose wavelength must fall in the so-called therapeutic window (500–900 nm), limited at shorter wavelengths by the absorption properties of several skin chromophores and at longer wavelengths by water absorption. The population of triplet states by intersystem crossing (ISC) and their consequent quenching mechanisms trigger a series of photoreactions that lead to biological damage. The occurrence of ISC strongly depends on the values of the spin–orbit coupling constants whose amplitude increases with an increased difference between the orbital natures involved in the transition [31]. Values previously computed for the approved PS Foscan [32,33] (5,10,15,20-tetrakis- (m-hydroxyphenyl) chlorin) are generally used as references for organic dyes. Once populated, direct photo-induced electron transfer processes can generate ROS [34,35] or even nitric-oxide radicals, able to react indiscriminately with DNA, lipids, and proteins [36] (Type I reactions), or they can promote an energy transfer mechanism to tissue oxygen, leading to the formation of the highly cytotoxic singlet oxygen (^3^Σ_g_ → ^1^Δ_g_) (Type II reaction). To some extent, triplet-deactivating pathways could be in competition among them. To cause irreversible destruction of neoplastic tissues, it is generally believed that the energy transfer should predominate over Type I, due to the relatively higher reactive property combined with the higher spatio-temporal control of the ^1^O_2_ release. Anyway, extremely powerful photosensitizers that show dual Type I/II activity have already been proposed, in which the combination of both mechanisms is evoked to explain the potent phototoxicity [23,26]. PS should possess specific requirements to be proposed as a therapeutic pro-drug, such as: (i) a red-shifted absorption wavelength to deeply penetrate human tissues; (ii) a singlet–triplet splitting (Δ_S-T_) high enough to excite oxygen and producing the singlet species (0.98 eV) [37]; and (iii) an efficient ISC mechanism that, in turn, increases the production of singlet oxygen. These crucial properties can be easily tuned by proper structural modification, including the use of heavy atoms to enhance ISC or choosing appropriate ligands to modify the photophysical and electronic properties. The search for metal-free and less-toxic solutions has led to consideration of more biocompatible main group elements to induce heavy atom effects [38,39,40]. Several intriguing investigations so far, carried out on chalcogen-modified nucleobases, demonstrated that exocyclic carbonyl oxygen replacement by either sulphur or selenium produce nucleobases able to generate singlet oxygen [41,42,43,44]. Moreover, stable RNA [45], DNA duplex [46,47], and G-quadruplex structures [47] have been found for selenium derivatives, which, moreover, exhibit improved photophysical properties due to red-shift absorption properties and faster ISC compared with their thio-counterparts. Such findings boost attention on chalcogen use to find new and appealing photosensitization agents. Indeed, besides nucleobases, Nguyen et al. [48] recently proposed thio-based naphthalimide dyes and their utility for PDT application in an hypoxia environment, and, almost at the same time, Xiao et al. reported a series of thio-based fluorophores starting from oxo-congeners, demonstrating that the thiocarbonyl derivatives exhibit a distinct batochromic shift, a significant fluorescence loss, and distinct singlet oxygen quantum yields that are missing in the oxygen counterparts, suggesting them as outstanding PS candidates for PDT [49]. Moreover, in both cases, an efficient population of long-lived, active triplet-excited states is evoked, consistent with the observed ability of sensitizing molecular oxygen.

Some of the proposed thio-compounds have been investigated in previous theoretical papers that confirm that the choice of chalcogens can be a promising strategy to achieve more suitable PDT agents while proposing an advance toward heavy-atom-free PSs [50,51].

**Figure 2:**
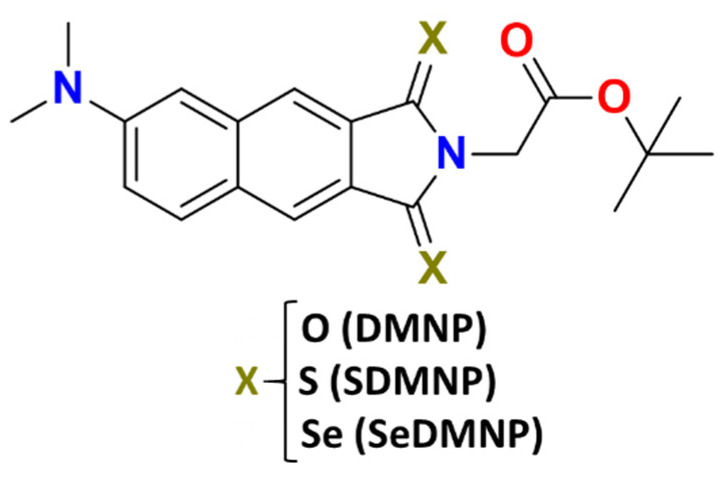
Molecular structures of DMNP, SDMNP, and SeDMNP, herein investigated, created with the free ChemSketch software available at www.acdlabs.com.

Herein, we investigated the effects of oxygen atom replacement by either S and Se on the photophysical properties of dimethylaminonaphthalimide dye [49] (DMNP, Figure 2) by means of density functional theory (DFT) and its time-dependent extension (TDDFT). Among the so-far proposed thio-carbonyl compounds [49], SDMNP has also been proposed for photoimmunotherapy due to the robust cytotoxicity exerted by its conjugate with trastuzumab, a monoclonal anti- body directed against HER2 [49], which enhances the interest of this dye. While the effects of sulphur have been experimentally examined [49], those relative to the seleno derivative have never been explored for this dye. Consequently, our investigation attempts to establish whether the Se-for-oxygen single-atom replacement can lead to an appealing candidate for PDT. At the same time, the possibility that both the chalcogen derivatives act as a dual Type I/II PS has been herein considered, along with the elucidation and characterization of absorption properties, singlet–triplet splitting, and the SOCs values. 

## 2. Results and Discussion

**Figure 3:**
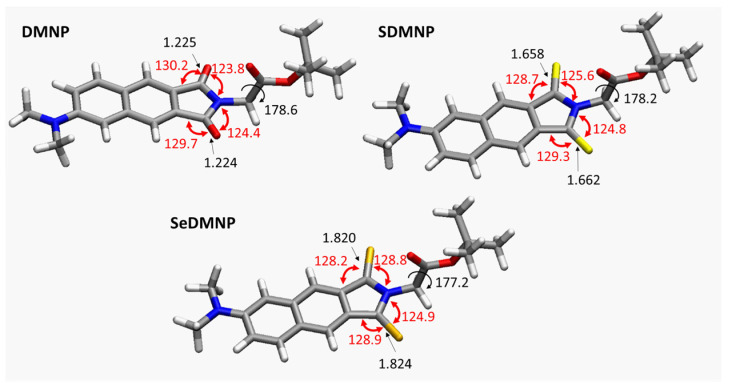
Optimized geometries of DMNP, SDMNP, and SeDMNP in DMSO environment at the B3LYP/6-31+G(d,p) level of theory. The Cartesian coordinates for the optimized structures are reported in Table S1.

The ground state conformations of the considered systems are depicted in Figure 3, together with the main geometrical parameters. Comparison between the obtained structures reveals that the sulphur- and selenium-for-oxygen substitution process does not affect significantly the geometrical parameters, with the exception of the C–X bond (X=O, S, Se), which is elongated along the chalcogen group. Indeed, the bond length increases from the value of 1.225 Å (X=O) to 1.658 Å (X=S), reaching the maximum length of 1.820 Å in the seleno derivative. Analogously, the X– C–N valence angle slightly increases in going from DMNP (123.8°) to SDMNP (125.6°) to SeDMNP (128.8°). The peripheral ester groups are perpendicular to the perfectly planar aromatic naphthalimide core in all cases, with dihedral angles of almost 180° (See Figure 3). 

**Figure 4:**
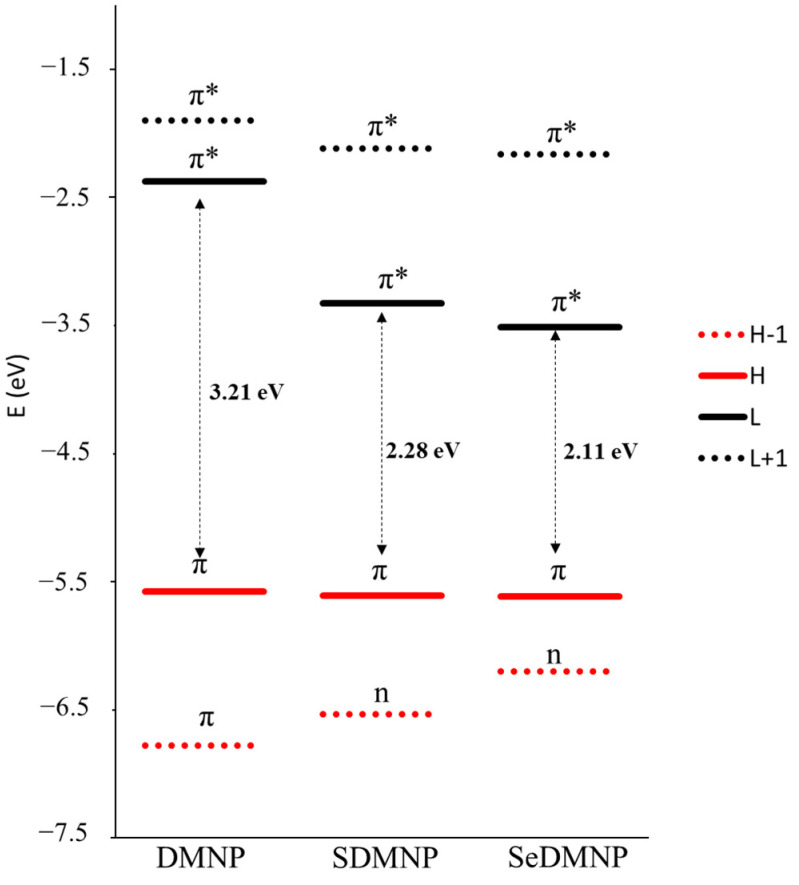
Gouterman frontier molecular orbital energies for DMNP, SDMNP, and SeDMNP.

Analysis of the four Gouterman frontier orbitals (Figure 4 and Figure S1) offers interesting details on the electronic change following single-atom replacement. Indeed, a significant drop in the energy of the LUMO orbital is obtained upon the introduction of sulfur and even more after the seleno-for-oxygen substitution. Inspection of the shape of the LUMO orbitals reveals a higher contribution of the chalcogens S and Se to the orbital composition (≈30%) compared with the low contribution of oxygen (9%). As a consequence, the stabilization of the LUMO for SDMNP and SeDMNP can be attributed to their hybrid π* character. An opposite trend can be observed for the HOMO-1 orbital, for which the increasing contribution of chalcogens to the orbital is proportional to the increasing of orbital energy and to the change in nature from π to n observed upon sulfur and seleno introduction.

**Figure 5:**
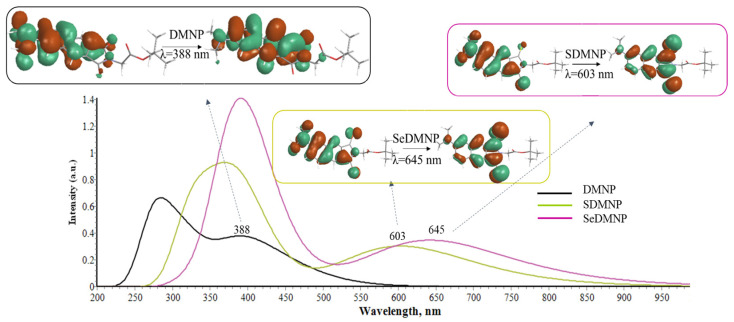
Computed Absorption Spectra of DMNP, SDMNP, and SeDMNP in DMSO environment at the B3LYP/6-31+G(d,p) level of theory and Natural Transition orbitals (NTOs) characterizing the bright lowest energy transition for each compound. See SI for more details.

Upon sulfur-for-oxygen replacement, the H→L lowest energy band undergoes a bathochromic shift, detected at 603 nm, and its π→π* nature has a considerable increase of density on the sulfur ligand. Analogously, the insertion of selenium further shifts the λ_max_ at a higher wavelength (645 nm), and the contribution of the chalcogen during the transition is unequivocally observed in the NTOs plot reported in Figure 5. As a consequence, SDMNP and SeDMNP reach a fully biocompatible region of the spectrum to be proposed as a PDT candidate. 

The possibility of populating the triplet states upon irradiation has also been considered. In the energy diagram of the main singlet and triplet states (See Figure S3), two lower-energy triplet states (T_1_, T_2_) lie below S1 for DMNP and SDMNP, and three (T_1_, T_2,_ and T_3_) for selenium derivative SeDMNP, and these can be considered in the possible S_1_→T_n_ intersystem crossing deactivation pathways. The higher triplet states, if populated, could easily decay to T1, whose energy is higher than that required to produce the ^1^Δ_g_ cytotoxic molecular oxygen (0.98 eV) for all the investigated compounds.

**Figure 6:**
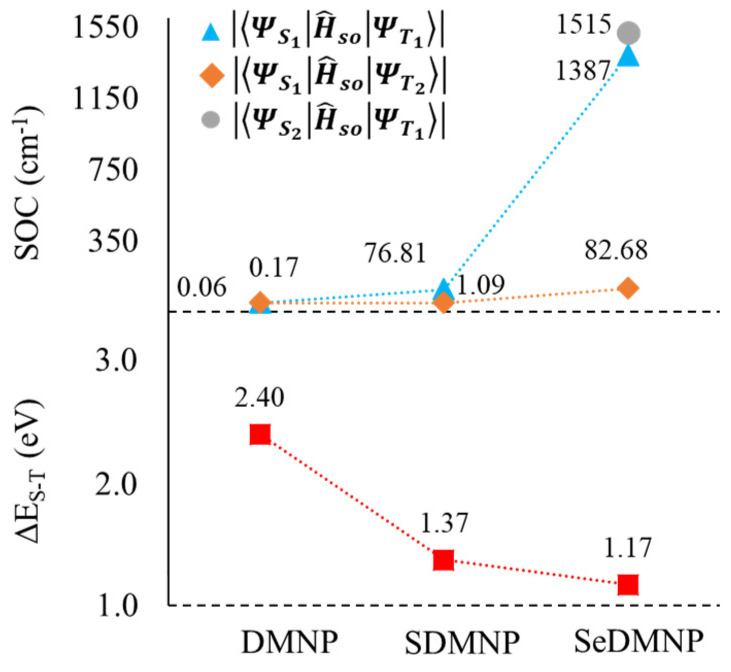
Computed SOC values (cm^−1^) and singlet–triplet splitting (Δ_S-T_, eV) for DMNP, SDMNP, and SeDMNP.

Figure 6 reports the computed SOC values for the S1-T1 and S2-T2 channels for all the derivatives and even the S2-T1 channel for SeDMNP, due to the close energy between the first two singlet excited states, together with the S0-T1 energy difference gap (Δ_S-T_) (See also Figure S3). The calculated SOCs for the considered channels clearly show that the oxygen replacement led to a significant increase in their values, suggesting a more efficient ISC for both S1-T1 and S1-T2 channels for SDMNP and SeDMNP. In the case of a seleno derivative, the S2-T1 channel has also been considered that shows a very high SOC (>1500 cm^−1^) and represents a further deactivation pathway. The computed values provide a rationale for the experimental reported singlet oxygen quantum yield values (Φ_Δ_) [49] that are negligible for DMNP but reach a higher value for sulfur- substituted species (Φ_Δ_ = 0.81) [49]. 

Besides the Type II mechanism, the occurrence of the electron transfer reaction leading to the highly reactive O_2_^.(−)^ species has also been verified. Two main mechanisms could lead to the formation of superoxide: a direct electron transfer from the photosensitizer (PS) to oxygen (1) or an electron transfer mechanism from a reduced form of the PS to O_2_ (2). The latter could be reduced by auto-ionization reactions (3) and (4), involving neighboring PS in the S0 or T1 states, according to the following reactions:

^3^Ps + ^3^O_2_ → Ps ^(+)•^ + O_2_^(−)•^ (1)

Ps^(−)•^ + ^3^O_2_ → ^1^Ps + O_2_^(−)•^ (2)

^3^Ps + ^1^Ps → Ps^(+)•^ + Ps^(−)•^ (3)

^3^Ps + ^3^Ps → Ps^(+)•^ + Ps^(−)•^ (4)

Knowledge of the vertical electron affinity (VEA) and ionization potentials (VIP) allow verification of the feasibility of the above-mentioned reactions. On the basis of the computed values summarized in Table 1 for DMNP, SDMNP, and SeDMNP, it can be deduced that the direct electron transfer to oxygen to produce superoxide (1) can be ruled out for sulfur and seleno derivatives, but it could occur for DMNP, with the sum of VEA (^3^O_2_) and VIP (^3^PS) slightly negative (0.10 eV), suggesting an energetically favorable reaction. 

The photosensitizer, once it has populated the lowest triplet state, could be reduced by auto-ionization through reaction (4), since the comparison between the VEA and VIP values considering both molecules in the triplet state (VEA and VIP ^3^PS) indicate an exothermic process for each derivative. This conclusion is not valid for the auto-ionization involving one of the PS reactants in the ground state (3) due to the positive summation value between VEA (^3^Ps) and VIP (^1^Ps). More importantly, the results show that once produced, the Ps^(−)•^ is able to undergo electron transfer to the oxygen with the production of superoxide (2) in DMNP and in the thio-derivative SDMNP. Indeed, comparing the electron affinity of dioxygen in water (−3.66 eV) with the corresponding values for both the above-mentioned compounds, the reaction is predicted to be favorable. Concerning the SeDMNP dye, the small difference between the electron affinity of molecular oxygen compared to VEA (^1^Ps) does not allow us to definitely exclude the occurrence of the reaction, since it is predicted to be exothermic by 0.5 eV. Moreover, the superoxide anion can itself act as a reducing agent for compounds in the triplet state, which may be indicative of an increased phototoxicity. Such evidence is supportive of a dual Type I/Type II activity of such compounds, further enhancing the importance of the use of chalcogens in dyes for PDT.

## 3. Computational Details

The DFT/B3LYP [52,53] method, as implemented in Gaussian 16 software [54], has been employed to carry on geometry optimizations and to compute excitation energies, by using the 6-31+G(d,p) basis set [45,46]. Dispersion corrections for nonbonding interactions were included by applying an atom pairwise additive scheme (DFT-D3) method [55]. Solvent dimethylsulfoxide (DMSO, ε = 46.82) effects were considered using the IEFPCM continuum solvation model [56]. Ref. [47] Excitation singlet and triplet energies were obtained in DMSO as vertical electronic excitations on the ground-state structures at the TD-DFT/B3LYP/6-31+G(d,p) level of theory. The Tamm-Dancoff approximation [57] has been used throughout to avoid triplet instabilities [58]. Singlet–triplet splittings (ΔE_S-T_) were computed at the same level of theory and compared with the previously computed gap for oxygen (0.90 eV) [59].

The SOCs values, defined as $SOC_{nm}={\sqrt{\underset{i}{\sum }\left|\langle \psi_{S_{n}}\left|{\hat{H}}_{SO}\right|\psi_{S}{}_{m}\rangle \right|}}_{i}^{2}\;\left(\mathrm{where}\;i=x,y,z\;;\;{\hat{H}}_{SO}=\mathrm{spin}–\mathrm{orbit}\;\mathrm{Hamiltonian}\right)$, were computed by using the atomic-mean field approximation [60] as implemented in the DALTON code [61] at the B3LYP/cc-pVDZ level of theory on the previously optimized structures. The accuracy of the chosen protocol was previously adopted to investigate the photophysical properties of several other organic photosensitizers [62,63,64,65,66].

SOCs and ΔE_S-T_ are both crucial parameters to estimate the occurrence of the non-radiative ISC mechanisms, considering that the KISC for the S_n_–T_m_ transition, in the Frank–Condon approximation and in the non-adiabatic regime, can be obtained by using the Fermi Golden rule expression [67]:

$$k_{ISC}^{nm}=\frac{2\pi }{\hbar }\langle \psi_{S_{n}}\left|{\hat{H}}_{SO}\right|\psi_{T}{{}_{m}\rangle }^{2}x\;FCWD$$

where ${\hat{H}}_{SO}$ is the spin–orbit Hamiltonian, and *FCWD* is the Franck–Condon weighted density of states that mainly depends on the Δ*E_S − T_* values [67]:

$$FCWD\propto \exp\left[-\frac{{\left(\Delta E_{S-T}\right)}^{2}}{4\lambda k_{B}T}\right]$$

## 4. Conclusions

DFT and TDDFT levels of theory have been used to determine the modulation of photophysical properties when sulfur and selenium atoms replace the oxygen in dimethylaminonaphthalimide dyes. Results show that the absorption Q band undergoes a significant red shift upon oxygen atom substitution, due to a drop in energy of the LUMO orbital caused by a higher contribution of chalcogen to the orbital. The reduced H–L gap determines a significant batochromic shift of the λ_max_, reaching a more biocompatible region of the spectrum. The spin–orbit coupling constants substantially increase when sulfur and selenium replaces oxygen, suggesting a more efficient ISC mechanism. From our data, SeDMN exhibits a more advantageous red-shifted absorption spectrum and faster ISC compared with its thio-counterpart, as suggested by the higher SOC values obtained for the three deactivation channels considered (T1→S1; T2→S1; T1→S2). All in all, the more feasible population of the triplet states, together with the Δ_S-T_ gap of appropriate energy to produce cytotoxic molecular oxygen (>0.98 eV), allow confirmation of the occurrence of Type II photoreactions for chalcogen-modified dyes. Concerning the electron transfer mechanisms with the production of superoxide, our results show that the auto-ionization mechanism (Type I) result feasible for all compounds and the reduced form of the triplet state could, in the case of DMNP and SDMNP, transfer an electron to oxygen with the production of superoxide. For Se-derivative, the process is predicted to be slightly exothermic. The results herein presented support the use of sulfur- and seleno-chalcogens to improve the photophysical properties of metal-free dyes for PDT.

## Figures and Tables

**Table 1:** B3LYP-D3/6-31+G(d,p) Vertical electron affinity (VEA) and vertical ionization potential (VIP) of DMNP, SDMNP, SeDMNP, and O_2_ in DMSO, in eV.

Cpds	VEA (S_0_)	VIP (S_0_)	VEA (T_1_)	VIP (T_1_)
DMNP	−2.48	5.42	−4.66	3.56
SDMNP	−3.51	5.43	−4.83	4.23
SeDMNP	−3.71	5.42	−4.84	4.39
O_2_			−3.66	

## Data Availability

The data presented in this study are available on request from the corresponding author.

## Supplementary Materials

The following supporting information can be downloaded at: https://www.mdpi.com/article/10.3390/molecules28073153/s1, Figure S1: Frontiers Molecular Orbital Plots; Figure S2: Main Vertical singlet and triplet excitation energies, λ (nm), ΔE (eV), oscillator strength f and Natural Transition Orbitals involved; Figure S3. Energy diagram of the main singlet and triplet states.
