# Supplementary material for: Sulphur- and Selenium-for-Oxygen Replacement as a Strategy to Obtain Dual Type I/Type II Photosensitizers for Photodynamic Therapy
Source: Molecules. 2023 Apr 1;28(7):3153. doi: 10.3390/molecules28073153 (PMC10095929; doi:10.3390/molecules28073153)
Supplement: Supplementary file 1 [file molecules-28-03153-s001.zip › molecules-2296292-supplementary.pdf]

-Supplementary Information-

Sulphur- and Selenium-for-Oxygen Replacement as a Strategy to Obtain Dual TypeI/TypeII Photosensitizers for Photodynamic Therapy

Mario Prejanò , Marta Erminia Alberto * , Bruna Clara De Simone, Tiziana Marino , Marirosa Toscano and Nino Russo *

Dipartimento di Chimica e Tecnologie Chimiche, Università della Calabria, 87036 Arcavacata di Rende, CS, Italy.

Figure S1: Frontiers Molecular Orbital Plots……………………………………………….pag.S2

Figure S2: Main Vertical singlet and triplet excitation energies, λ (nm), ΔE (eV), oscillator strength f and Natural Transition Orbitals involved…………………………………………………pag.S3

Figure S3. Energy diagram of the main singlet and triplet states………………………….….pag.S5

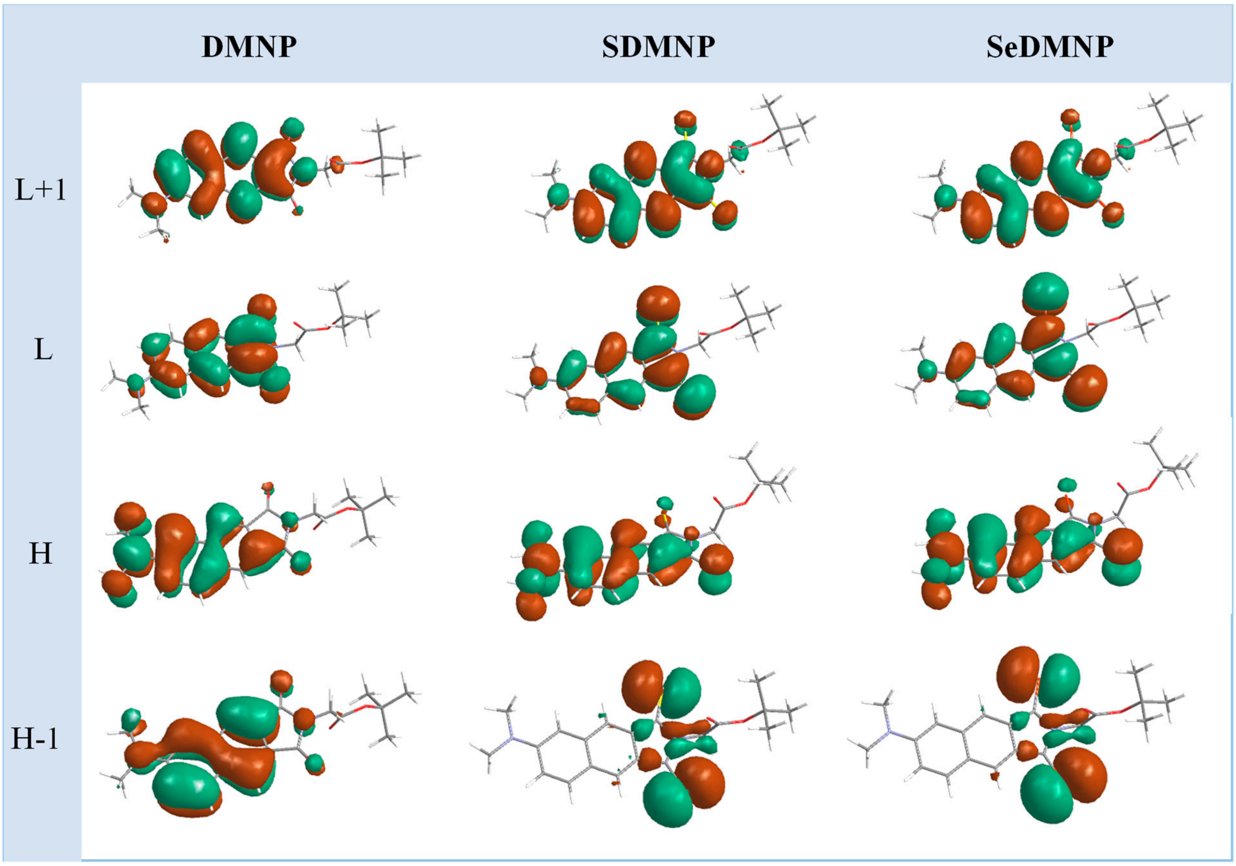

Figure S1: Frontiers Molecular Orbital Plots

**State**	**Composition**	**λ**	**ΔE**	**f**	**Natural Transition Orbitals (NTOs)**
			DMNP		
**S_1**	H→L	449	2.76	0.135	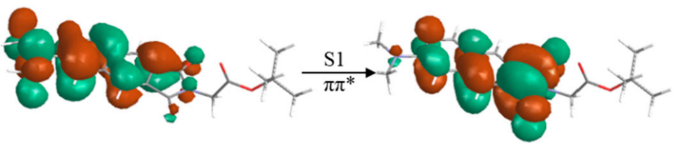
**S_2**	H→L+1	388	3.20	0.304	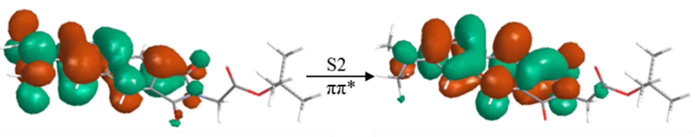
**T_1**	H→L	548	2.26	/	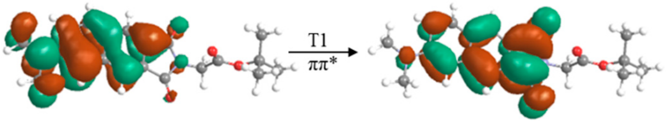
**T2**	H-1→L	493	2.51	/	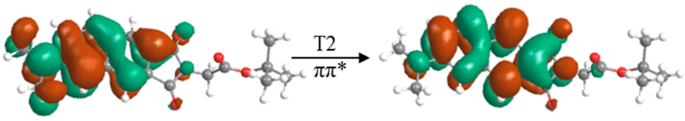
			SDMNP		
**S_1**	H→L	603	2.05	0.303	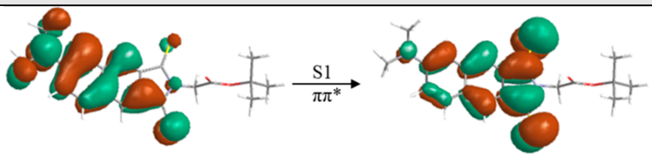
**T_1**	H→L	867	1.43	/	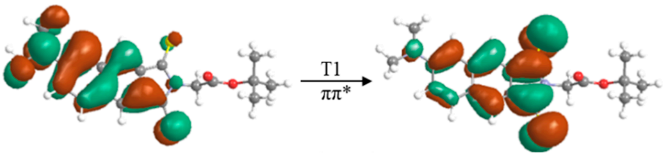
**T2**	H-1→L	626	1.98	/	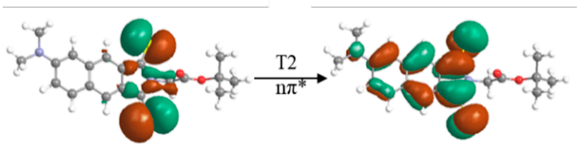
			**SeDMNP**		

**S_1**	H-1→L	682	1.82	0.000	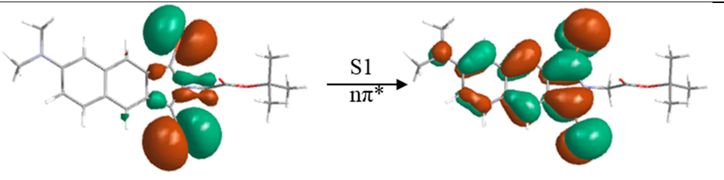
**S2**	H→L	645	1.92	0.333	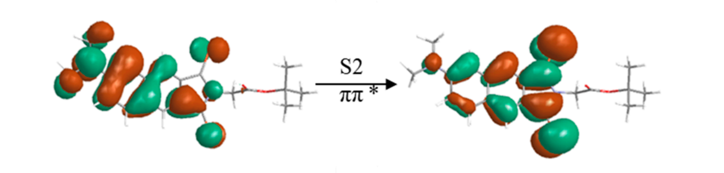
**T_1**	H→L	1001	1.24	/	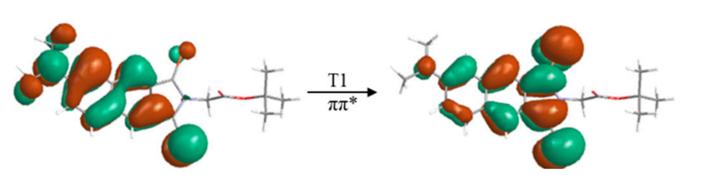
**T2**	H-1→L	787	1.58	/	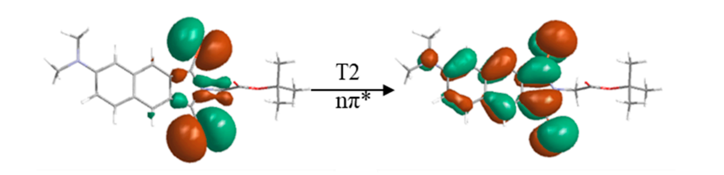

Figure S2: Main Vertical singlet and triplet excitation energies, λ (nm), ΔE (eV), oscillator strength f and Natural Transition Orbitals involved, for DMNP, SDMNP and SeDMNP in DMSO at TD-DFT/B3LYP/6-31+G(d,p) level of theory.

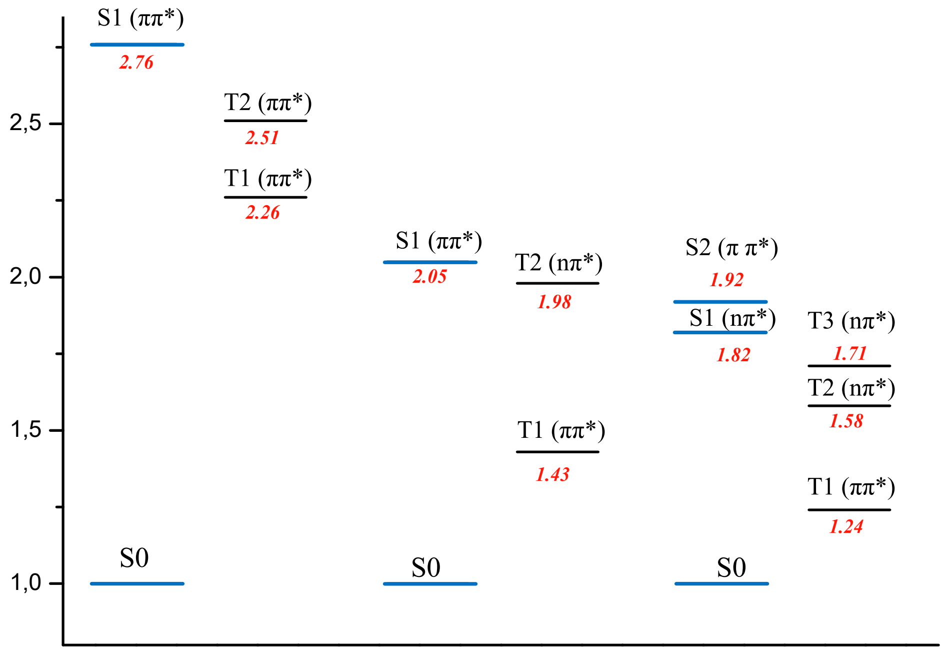

Figure S3. Energy diagram of the main singlet and triplet states.
